# Actin’s functional “switch”: Constraining C-terminal conformational flexibility disrupts functionally important communication networks

**DOI:** 10.1016/j.jbc.2026.111446

**Published:** 2026-04-09

**Authors:** Karl E. Steffensen, John F. Dawson

**Affiliations:** Department of Molecular and Cellular Biology, University of Guelph, Guelph, Ontario, Canada

**Keywords:** actin, actomyosin, phenylenebismaleimide, molecular dynamics, computational modeling, protein structure, allosteric communication

## Abstract

Actin’s C terminus contributes to interactions with actin-binding proteins (ABPs) in cellular processes. Historical resolution constraints have prevented a complete understanding of how actin’s C terminus mediates actin’s interactions, but evidence suggests that it undergoes conformational shifts upon ABP binding. Actin’s C terminus also shifts in response to actin’s nucleotide state, guiding nucleotide-dependent ABP binding. Previously, we proposed that C-terminal shifts regulate actin’s functions through allosteric communication networks. To further examine the role of C-terminal shifts in actin communication networks and understand how C-terminal modifications influence actin’s functional state, we utilized *in silico* modeling of actin filaments crosslinked by *N*,*N*′-*para*-phenylenebismaleimide (PBM). Our modeling examined how constraining C-terminal flexibility with PBM crosslinks affects the conformation of distant structural elements and shifts internal communication networks critical for actin’s functions. We present evidence that disruption of the stabilizing F375–R116 interaction by PBM-crosslinks reshapes allosteric communication networks through the protomer, altering nucleotide cleft architecture and nucleotide dynamics. We therefore propose that actin’s C terminus acts as a nexus for actin structural changes, shifting in response to stimuli, and propagating changes that allow actin to adopt different functional conformations that influence ABP binding.

Actin contributes to essential cellular processes through interactions with actin-binding proteins (ABPs) (1, 2, 3), many of which utilize actin’s highly flexible C terminus (residues 370–375) (4, 5). After polymerizing from monomeric globular actin (G-actin), two-stranded filamentous actin (F-actin) is stabilized through interactions between actin’s C terminus and the DNaseI binding loop (D-loop, residues 38–53) of a neighboring protomer, forming an interprotomer interface that acts as the main ABP-binding site in F-actin (6, 7, 8).

Changes in C-terminal position, structure, and flexibility have been connected to altered actin properties and ABP binding (9, 10, 11), and past studies sought to understand the role of actin’s C terminus in actin–ABP interactions. Due to its inherent flexibility, many studies examined the impact of constraining C-terminal flexibility with chemical crosslinks (12, 13, 14, 15). For example, crosslinking F-actin with *N*,*N*′-*para*-phenylenebismaleimide (PBM) was found to have an uncoupling effect on the activity of force-generating myosin following actin binding (15). In short, myosin bound PBM–actin filaments and generated ATPase activity, but subsequent force generation was impeded. Further studies with fluorescent labels conjugated to actin residue C374 observed changes in fluorescence upon myosin binding (16, 17), suggestive of conformational shifts at the C terminus. It was therefore proposed that actin’s C terminus undergoes a conformational shift upon myosin binding, with PBM-crosslinks preventing such a shift and restricting force generation. Subsequent studies focused on actin’s C terminus, however, were impeded due to resolution constraints (11), preventing further characterization of C-terminal roles within actin–ABP interactions.

Previously, we reviewed the role of actin’s C terminus in regulating functional interactions (18), finding that C-terminal flexibility dictates actin polymerization and ATPase activity, overall filament structure and stability, as well as ABP binding. We therefore proposed several structural elements that might be connected to the C terminus *via* internal communication networks, with actin C-terminal flexibility guiding changes in distant regions for the regulation of actin’s functions. For example, recent high-resolution structures have identified a direct communication pathway between actin’s C terminus and its nucleotide-binding cleft (7). Actin’s C terminus might therefore respond to changes in actin’s nucleotide state, directing nucleotide-dependent binding of ABPs. Understanding how actin’s C terminus participates in functional communication networks is important for understanding fundamental cellular processes such as actomyosin force generation, as well as structural shifts because of disease-causing amino acid substitutions.

We hypothesize that actin’s C terminus participates in two-way communication networks, responding to various stimuli that allow it to regulate functionally important shifts in structural properties and conformations. To understand how C-terminal flexibility might regulate actin’s structure and functions through allosteric communication networks, we conducted extensive molecular dynamics (MD) simulations of actin filaments with C termini constrained by PBM-crosslinks. To study actin communication networks, we wanted to model actin with altered C termini and characterize the impacts. We selected PBM–actin as the focus of this study for several reasons: the wealth of previous *in vitro* functional data; the ability to remove C-terminal flexibility and induce a conformational change while maintaining filament structural integrity; and the fact that PBM–actin can form productive interactions with ABPs, unlike many actin variants. Finally, actin’s C terminus is connected to distant regions *via* the R116–F375 interaction. As R116 interacts with the backbone of F375, mutagenesis of C-terminal residues may not disrupt this interaction enough to identify changes throughout actin’s structure. PBM–actin is therefore a strong candidate to understand C-terminal connections within actin’s structure. It should be noted that we are not looking to predict the exact structures of PBM actin filaments, rather we seek to understand how PBM might alter filament dynamics *via* actin’s C terminus, providing further context to previous *in vitro* experimental data and improving our understanding of actin’s role in cellular interactions.

We find that the introduction of PBM-crosslinks did not result in large-scale structural rearrangements of F-actin. Rather, we observed that structural rearrangements were highly localized to the C terminus, nucleotide cleft, and the D-loop. Further, we observed that PBM–actin loses many internal communication pathways, which instead shift to the periphery of the structure. As many of the altered communication networks pass through actin’s nucleotide-binding cleft, we predict that C-terminal changes are connected to the properties and conformations of residues regulating actin’s nucleotide dynamics. Taken together with past studies, we propose that actin’s C terminus acts as a nexus for functionally important communication networks, with its inherent flexibility inducing actin structural changes that regulate its interactions and functions. Further studies examining C-terminal allosteric networks can uncover key details of actin–ABP interactions, improving our understanding of essential cellular processes.

## Results

Actin’s C terminus has been connected to actin structural changes and functions (18), with previous studies observing functional changes from crosslinking actin’s C terminus (15). Due to a lack of structural models, there was no clear mechanism driving these changes. As actin’s C terminus is involved in allosteric communication (18), we utilized MD simulations (Tables 1, and 2) of PBM–actin to observe shifts in communication pathways. Our analyses focused on three aspects: identifying structural changes correlating with C-terminal changes; determining how allosteric communication shifts with C-terminal conformation; and understanding the functional implications of altering actin’s C terminus.

**Table 1:** Simulation setup

System	Number of protomers	PBM crosslinks (protomer)	Protomers for analysis	Simulation time
PBM-dimer	6	1. C374 (PD1)-K191 (PD2) 2. C374 (PD3)-K191 (PD4) 3. C374 (PD5)-K191 (PD6)	PD3, PD4	2 × 200 ns
CD	6	N/A	CD3, CD4	2 × 200 ns
PT	9	1. C374 (PD1-K191 (PD2) 2. C374 (PD2)-K191 (PD3) 3. C374 (PD4)-K191 (PD5) 4. C374 (PD5)-K191 (PD6) 5. C374 (PD7)-K191 (PD8) 6. C374 (PD8)-K191 (PD9)	PT4, PT5, PT6	2 × 200 ns
CT	9	N/A	CT4, CT5, CT6	2 × 200 ns

N/A, not available.

The systems simulated are listed in the table, including their designation based on PBM status and number of crosslinks, the number of protomers making up a filament, the numbers of crosslinks and residues in each protomer that have been crosslinked, the protomers chosen for analysis, as well as the number of replicates and simulation time per replicate.

**Table 2:** System details

System	Box dimensions (nm)	Box volume (nm_3_)	# Atoms				
			Total	Protein	Protein + ligands	PBM	Water + ions
WT 6 subs	23.11985 23.11985 23.11985	12,357.63354	1,218,240	34,980	35,250	N/A	1,182,990
WT 9 subs	31.56887 31.56887 31.56887	31,461.33232	3,088,740	52,470	52,875	N/A	3,035,865
2er 6 subs	23.11985 23.11985 23.11985	12,357.63354	1,218,354	34,971	35,319	78	1,182,957
3er 9 subs	31.56887 31.56887 31.56887	31,461.33232	3,088,878	52,452	53,013	156	3,035,709

N/A, not available.

For each simulation system, the details of how each system was built in GROMACS are listed in the table. Box dimensions and volume were generated automatically by the GROMACS module *gmx editconf*, maintaining a 1 nm distance between the protein and the edge of the box at all times.

### Structural properties

**Figure 1:**
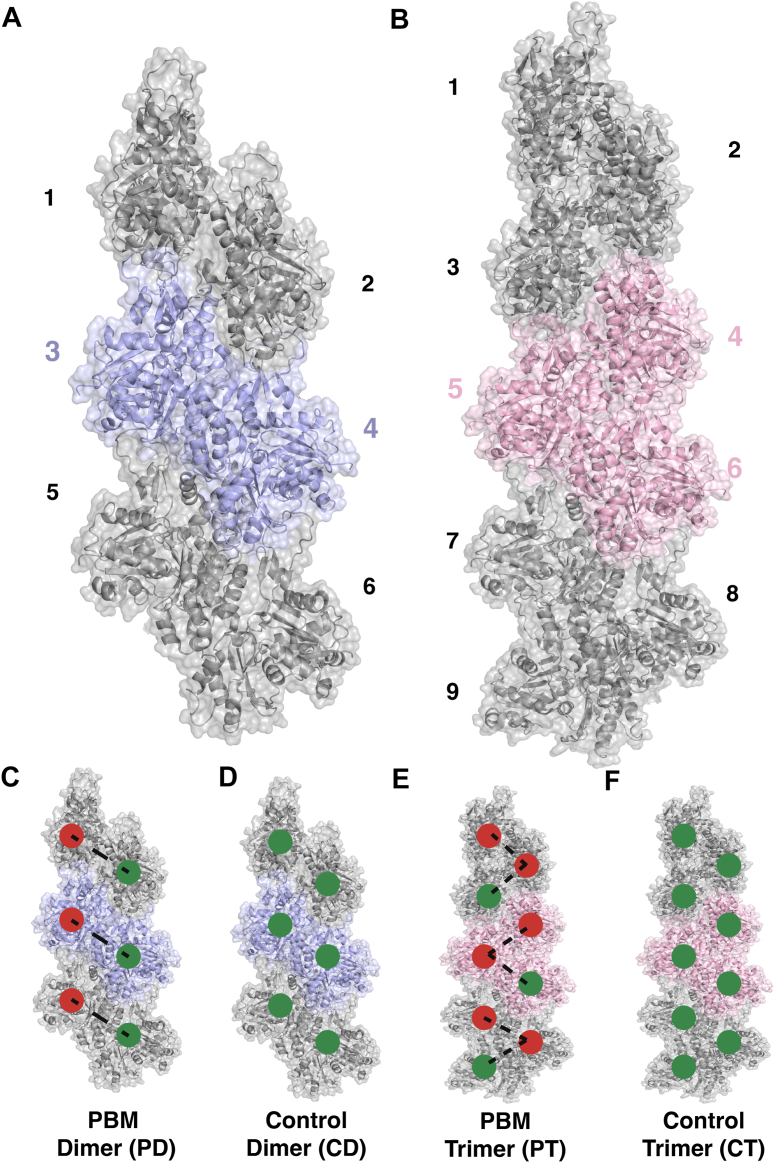
**PBM simulation setup.** Molecular dynamics (MD) simulations were conducted for actin filaments, with and without PBM crosslinks, to investigate the impacts of C-terminal modifications. PBM crosslinks bridge C374 of one protomer to K191 of a cross-strand neighbor, with crosslinking reactions occurring randomly to form oligomers of various sizes (monomer, dimer, trimer, etc.) within the filament, each with different numbers of PBM crosslinks. *A* and *B*, to study the behaviors of different oligomers, filaments composed of six (*A*) and nine (*B*) protomers were formed. *C*, PBM crosslinks were introduced to form a filament composed of three PBM-dimers, where C374 of one protomer is linked to K191 of its cross-strand neighbor. Protomers with crosslinked C termini are denoted by *red circles*, whereas protomers with uncrosslinked C termini (*i.e.*, crosslinked K191) are denoted by *green circles*. *Dashed black lines* represent PBM crosslinks. *D*, a control filament composed of six protomers without PBM crosslinks was also formed. *E*, PBM crosslinks were introduced to form a filament composed of three trimers. Protomers with crosslinked C termini are denoted with *red circles*, whereas protomers with uncrosslinked C termini (*i.e.*, crosslinked K191) are denoted by *green circles*. *Dashed black lines* represent PBM crosslinks. *F*, a control filament composed of nine protomers without PBM crosslinks was also formed. All filaments shown were generated from PDB 8A2S. PBM, *N*,*N*′-*para*-phenylenebismaleimide; PDB, Protein Data Bank.

**Figure 2:**
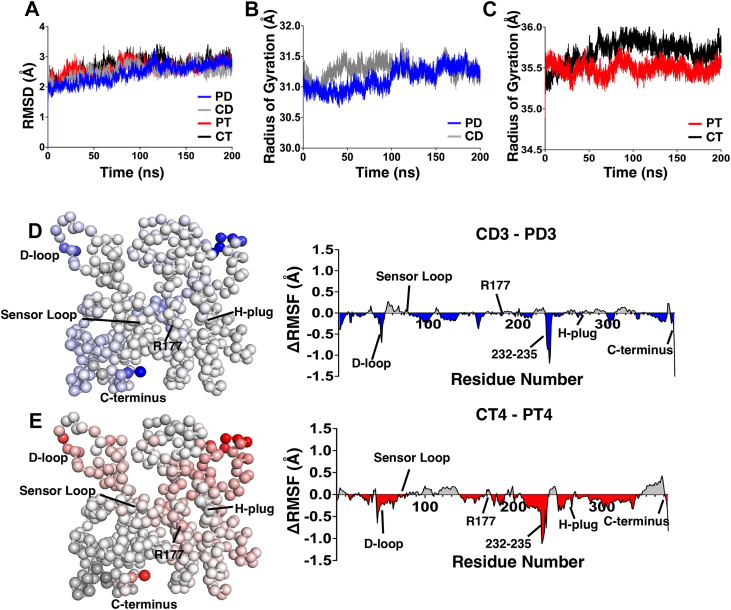
**Structural properties.** Protomers within the central oligomer containing crosslinked C termini were isolated and analyzed to determine the impacts of C-terminal alterations. *A*, the average RMSD values across both replicates were plotted for PBM-dimer (PD, *blue*), control-dimer (CD, *gray*), PBM-trimer (PT, *red*), and control-trimer (CT, *black*), showing no difference in average RMSD over time. *B*, average radius of gyration (Rg) across both replicates was plotted for PD (*blue*) and CD (*gray*) systems, showing no differences. *C*, average Rg across both replicates for PT (*red*) and CT (*black*) systems, with a slight decrease in Rg in the PBM-crosslinked protomer. *D*, ΔRMSF values (control – PBM) were calculated for central dimer protomers (protomer 3 as shown in Fig. 1) and projected onto the structure of an actin protomer. Negative values (PBM > control, colored *blue*) indicate increased residue flexibility in the PBM structure, whereas positive values (control > PBM, colored *black*) indicate increased flexibility in the control structure. *E*, ΔRMSF values (control – PBM) were calculated for central trimer protomers (protomer 4 as shown in Fig. 1) and projected onto the structure of an actin protomer. Negative values (PBM > control, colored *red*) indicate increased residue flexibility in the PBM structure, whereas positive values (control > PBM, colored *black*) indicate increased flexibility in the control structure. PBM, *N*,*N*′-*para*-phenylenebismaleimide.

Structural properties of the PBM-crosslinked and PBM-free control systems were compared to identify widespread structural changes. To allow a direct comparison of the impacts of PBM-crosslinks, properties were analyzed for central protomers with crosslinked C374 (PD3, PT4) as well as their respective PBM-free control counterparts (CD3, CT4) (Fig. 1, Tables 1, and 2). Average RMSD was plotted for each system with no differences observed (Fig. 2*A*), whereas the average radius of gyration (Rg) suggests that protomer PT4 is slightly more compact than CT4 (Fig. 2, *B* and *C*).

Root mean square fluctuation (RMSF), a measure of average residue flexibility, was determined for individual residues, and the difference in RMSF values between PBM and control systems (ΔRMSF) was plotted (Fig. 2, *D* and *E*). RMSF values for PBM-crosslinked protomers (PD3, PT4) were subtracted from their PBM-free counterparts (CD3, CT4), with a negative value indicating greater RMSF in the PBM-crosslinked structures relative to PBM-free controls. ΔRMSF values were also projected onto a protomer of actin isolated from the Protein Data Bank (PDB ID: 8A2S). In the dimer structure (Fig. 2*D*), blue residues indicate a negative ΔRMSF (PD3 > CD3), and black residues indicate a positive ΔRMSF (CD3 > PD3). In the trimer structure (Fig. 2*E*), red residues indicate a negative ΔRMSF (PT4 > CT4), whereas black residues indicate a positive ΔRMSF (CT4 > PT4). In both systems, white residues indicate no difference in RMSF values. PBM-crosslinked systems had greater RMSF values relative to control structures at residue F375, the outside of the D-loop (residues 45–51), and residues 232 to 235 at the top of subdomain (SD) 4.

RMSF values were also plotted for cross-strand protomers with crosslinked K191 (Fig. S1, chains PD5, CD5, PT6, and CT6).

### Structural changes

**Figure 3:**
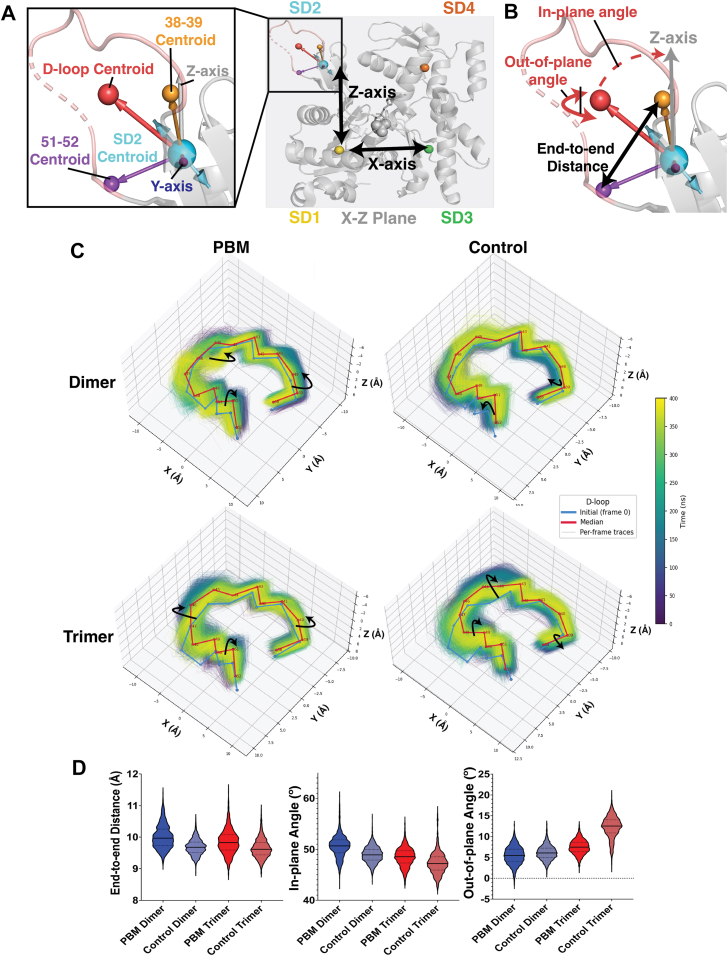
**Changes to the D-loop.** Shifts in D-loop positioning over time were measured. *A*, centroids (mean position of all residues) were calculated for each subdomain (SD1—*yellow*, SD2—*cyan*, SD3—*green*, and SD4—*orange*) as well as the D-loop (*red*, residues 38–52). An axis system was defined, with the *X*-axis connecting centroids of SDs 1 and 3 (*left*/*right*), the *Z*-axis perpendicular to the *X*-axis in the direction of SD2 (*up*/*down*), and the *Y*-axis perpendicular to these axes (*in*/*out* of the screen). Within this axis system, the XZ plane was defined to represent the “face” of the actin protomer. One subunit of PDB 8A2S was used to visualize the axis system. *B*, D-loop shifts were measured within this plane (“in-plane” angle, measured as the angle *left*/*right* of the *Z*-axis) as well as out of this plane (“out-of-plane” angle, measured as the angle forward/backward of the *Z*-axis). *C*, the D-loop coordinates were plotted in 3-dimensional space for each time point (0–400 ns representing duplicate 200 ns trajectories) in the simulations and colored based on time, along with the initial D-loop position (colored *blue*) and median D-loop position (colored *red*). The D-loop centroid was defined as the origin (0, 0, 0), and all D-loop coordinates were recalculated relative to the centroid position. *D*, D-loop positional changes were quantified. For each time point, the end-to-end distance (the distance from the centroid of residues 38–39 to the centroid of residues 51–52), in-plane angle, and out-of-plane angle were measured for each system. Within the violin plots, mean values are represented by *solid lines* and quartiles by *dotted lines*. D-loop, DNaseI binding loop; PDB, Protein Data Bank.

Next, we identified structural elements with large conformational differences. Due to differences in RMSF values at the D-loop, shifts in D-loop position over the simulation were measured (Fig. 3). For both replicates, the centroid of the D-loop (defined as the mean position of all D-loop residues) was identified at each time point. The 3D coordinates of each D-loop residue were plotted relative to the centroid for each time point (Fig. 3*C*). For both control systems, residues 38 to 40 exhibited “inwards” positioning toward the centroid, whereas these residues exhibited “outwards” motions away from the centroid in PBM systems. Further, residues 45 to 52 exhibited wider ranges of positions in PBM systems relative to control systems. Changes in D-loop positioning were quantified, with the D-loop width (measured as the distance from the centroid of residues 38–39 to the centroid of residues 51–52) exhibiting a larger variation and mean distance in PBM systems than in the control. Furthermore, an axis system was defined at each time point, where the *X*-axis connects the centroid of SDs 1 and 3, with the *Z*-axis defined perpendicular to this axis in the direction of SD2 (Fig. 3*A*). D-loop positioning relative to this axis system was then measured over time. The angle of the D-loop centroid to the *Z*-axis within the XZ-plane (“in-plane” angle or angle “left or right” of the *Z*-axis) as well as the angle of the D-loop along the *Y*-axis (“out-of-plane” angle or angle “forwards/backwards” of the *Z*-axis) was measured (Fig. 3*B*). Both PBM systems exhibited increased average in-plane and decreased average out-of-plane angles relative to their control counterparts, suggesting a shift in D-loop positioning in PBM systems.

**Figure 4:**
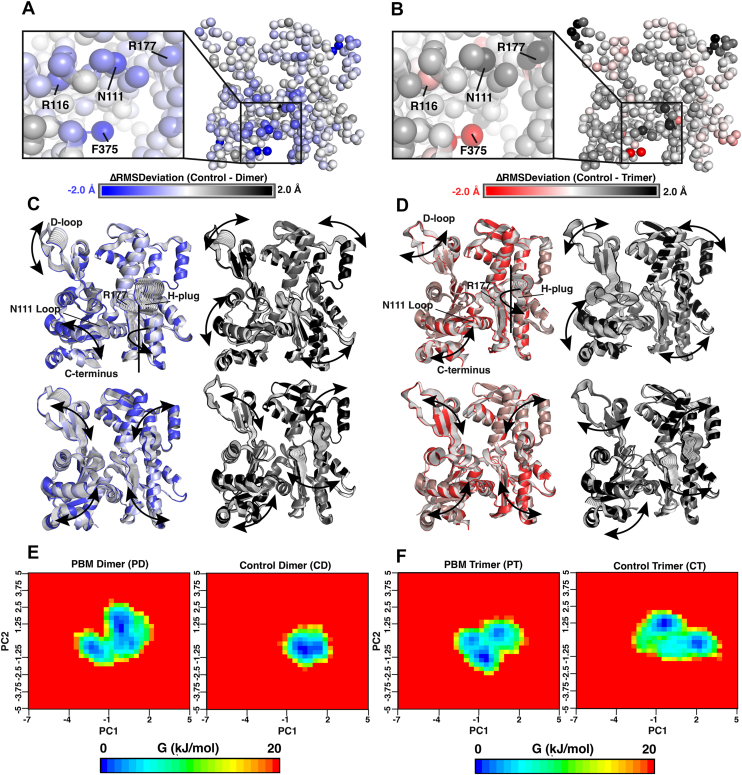
**Visualization of structural changes.** Differences in the structure of actin between PBM-crosslinked and uncrosslinked protomers were analyzed. *A*, through cluster analysis, the 20 most common conformations sampled by PD and CD systems were isolated. For each residue, the RMSD, a measure of conformational difference between two structures, was calculated for each cluster relative to the starting structure. Difference in RMSD (control – PBM) was calculated for central dimer protomers (PD3, CD3) and projected onto the structure. Negative values (PBM > control, colored *blue*) indicate a greater average RMSD (and greater difference from starting conformations) within PBM clusters, whereas positive values (control > PBM, colored *black*) indicate a greater RMSD within control clusters. Residue alpha-carbons are represented as *spheres*, whereas the backbone is shown as a *ribbon*. *B*, difference in RMSD (control – PBM) was calculated for central trimer protomers (PT3, CT3) and projected onto the structure. Negative values (PBM > control, colored *red*) indicate a greater average RMSD within PBM clusters, whereas positive values (control > PBM, colored *black*) indicate a greater RMSD within control clusters. Residue alpha-carbons are represented as *spheres*, whereas the backbone is shown as a *ribbon*. *C* and *D*, principal component analyses (PCA) identified the dominant motions within each system. The extremes of these dominant motions were projected onto the structure of a protomer central to the filament (PD—*blue*, CD—*black*, PT—*red*, and CT—*black*). *E* and *F*, Gibbs free energy landscapes (FELs) were calculated, showing coordinates along the two main principal components, PC1 and PC2, containing frequently sampled conformations. *Blue areas* represent high sampling frequency, indicative of favorable, low-energy conformations. *Red areas* represent low sampling frequency, indicative of less favorable, higher energy conformations. CD, control dimer; PBM, *N*,*N*′-*para*-phenylenebismaleimide; PD, PBM-dimer.

The 20 most common conformations in each system were isolated through cluster analyses. For each cluster, the difference in each residue relative to the starting structure (RMSD) was calculated and averaged. Net RMSD differences (control RMSD – PBM RMSD) were projected onto an actin protomer (Fig. 4, *A* and *B*). Large differences between PBM and control systems are localized to the C terminus, nucleotide cleft, and D-loop regions. In addition, structural elements connecting the C terminus to the nucleotide cleft, such as the N111 loop (residues 107–112), sensor loop (residues 72–77), and Pi-gate (residue 177), exhibit differences between PBM and control systems.

Principal component analyses (PCA) isolated the main vectors of motion for each system. The extreme motions along the two main principal components (PC1, PC2) were projected onto the structure, visualizing the range of structural changes sampled (Fig. 4, *C* and *D*). Along PC1, all systems experienced similar pivoting of SDs 1 and 2, with similar magnitude shifts in the D-loop between PBM and control systems. PBM-crosslinked structures, however, exhibited a hinge-like rotation of SDs 3 and 4 away from SDs 1 and 2 that alters the shape and accessibility of the nucleotide-binding cleft region. For both systems, increased ranges of motion are seen at Pi-gate (residues 173–179) as well as the hydrophobic plug (H-plug, residues 262–274). Control dimer (CD; Fig. 4*C*) exhibited an overall decreased range relative to PBM-dimer (PD), whereas control trimer (CT; Fig. 4*D*) exhibited increased ranges of motion at the interface of SDs 1 and 2. Along PC2, all systems showed similar overall motions, though both PBM structures exhibited a larger range of motion in SD2.

Next, combined PBM and control trajectories for each system (PD + CD, PBM-trimer [PT] + CT) were used to identify common vectors of motion and Gibbs free energy landscapes (FELs) were calculated for each system along these vectors (Fig. 4, *E* and *F*). CD structures are represented by a single energy basin compared with two in PD (Fig. 4*E*). CT structures are represented by two minimums, compared with three in PT (Fig. 4*F*). Both PBM structures contain one energy minimum with coordinates overlapping a minimum in the control landscapes, suggesting similar conformations are sampled between these systems. Additional energy minima in PBM structures, however, suggest additional sampling of unique conformations that are inaccessible to control systems.

The differences in actin domain angles were also measured, following methods used by Oda *et al.* (6), observing very little difference in the tilt of actin’s domains (Fig. S2).

### Allosteric communication

Actin’s C terminus is connected to distant structural elements through internal communication networks (10, 19, 20). We observed that the largest structural changes induced by PBM-crosslinks are localized to the C terminus and structural elements with known connections to the C terminus. We therefore wanted to understand how PBM-induced changes at the C terminus might propagate through actin’s structure.

**Figure 5:**
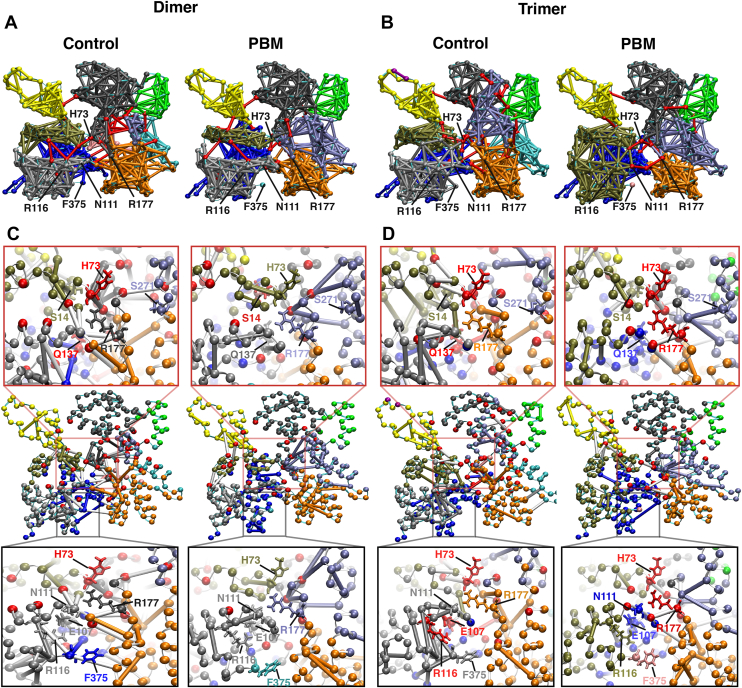
**Dynamic network analysis.** A dynamic network analysis was conducted for each system, identifying groups of residues with correlated motions (referred to as communities). Each community was projected onto the structure of a protomer and assigned a unique color, with residues represented by *spheres* and connections between residues by *lines*. *Red residues* and *connections* are critical nodes, which represent residues or connections that are important for the entire network. *A*, communities were compared between PD3 and CD3 protomers. *B*, communities were compared between PT4 and CT4 protomers. *C* and *D*, connections unique to each system were shown on the structure of protomers PD3, CD3, PT4, and CT4. A closer visualization of unique connections around the nucleotide cleft (*red box*) and C terminus (*black box*) is provided. Important nucleotide-regulating residues Q137, R177, S14, and H73, as well as important residues in the vicinity of the C terminus, F375, R116, E107, N111, and R177, are highlighted. CD, control dimer; PD, PBM-dimer.

Dynamic network analyses identified groups of residues (communities) with correlated motions (Fig. 5). Relative to control, PD exhibits reduced connectivity between the pathogenic helix (residues 112–126) and the helix directly above it (residues 78–91), with greater SD3 connectivity (Fig. 5*A*). In PT, SD1 acts as one large community, exhibiting greater connectivity relative to the control structure (Fig. 5*B*). Both PBM systems contained fewer connections across the nucleotide-binding cleft critical nodes (colored red). Control systems also contained more unique connections relative to PBM (Fig. 5, *C* and *D*), mostly concentrated around the nucleotide-binding cleft.

**Figure 6:**
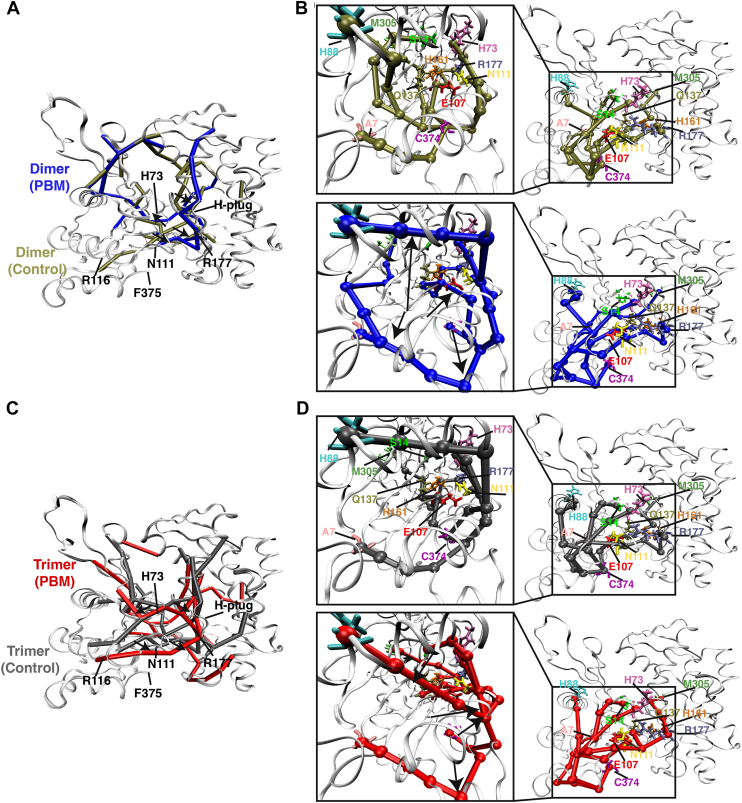
**Critical communication pathways.***A*, dynamic network analysis provided “betweenness” values for each residue–residue connection, which is a measure of how many communication pathways pass through that connection. Connections with betweenness values greater than 5000 are shown for protomers PD3 (*blue*) and CD3 (*tan*). *B*, suboptimal paths, representing shortest routes of communication between two nodes, were calculated between residue C374 and residues in key functional regions. The chosen residues represent connections between the C terminus and the nucleotide-binding cleft (E107, Q137), residues regulating nucleotide dynamics (Q137, H73, S14, H161, and M305) and phosphate release (N111, R177), as well as the N terminus (A7) and the interface between SDs 1 and 2 (H88). As no pathways existed between F375 and these residues in the PBM structures, suboptimal paths from C374 were calculated. The shortest calculated paths between C374 (*magenta*) and chosen residues (A7—*light pink*, S14—*lime green*, H73—*dark pink*, H88—*cyan*, E107—*red*, N111—*yellow*, Q137—*tan*, H161—*orange*, R177—*purple*, and M305—*dark green*) are shown for protomers CD3 (*tan*) and PD3 (*blue*), with highlighted residue side chains represented as *sticks*. *Black arrows* indicate how the shortest suboptimal paths shift from CD3 to PD3. *C*, connections with betweenness values greater than 5000 are shown for protomer PT4 (*red*) and CT4 (*gray*). *D*, the shortest calculated suboptimal paths between C374 (*magenta*) and chosen residues (A7—*light pink*, S14—*lime green*, H73—*dark pink*, H88—*cyan*, E107—*red*, N111—*yellow*, Q137—*tan*, H161—*orange*, R177—*purple*, and M305—*dark green*) are shown for protomers CT4 (*gray*) and PT4 (*red*), with highlighted residue side chains represented as *sticks*. *Black arrows* indicate how the shortest suboptimal paths shift from CT4 to PT4. CD, control dimer; PD, PBM-dimer; SD, subdomain.

Building on dynamic network analyses, the most important connections in each network were identified from the number of pathways mediated by a given connection (Fig. 6, *A* and *C*). Critical pathways in control structures passed through the center of the protomer from the C terminus through the nucleotide-binding cleft, connecting all four SDs. Critical connections in the PBM structures reside on the periphery rather than the interior of the protomer.

Finally, the most direct pathways between two residues (suboptimal paths) were calculated from the C terminus to residues in critical areas of the structure (Fig. 6, *B* and *D*). In PD, no pathway exists between C374 and S14. Further, pathways are shifted to the periphery, taking indirect routes between residue pairs. For example, C374–A7 proceeds internally *via* three mediating residues in CD, while traveling the length of the C terminus and bottom of SD1 *via* six mediating residues in the PD. In addition, cross-domain pathways from C374 to H88 and M305 take internal paths in CD through the nucleotide-binding cleft. In PD, these pathways instead pass through the pathogenic helix before moving up toward either H88 or the sensor loop and across toward M305. The C374–H88 and C374–M305 pathways are mediated by four and five residues, respectively, in CD compared with 8 and 11 residues in PD. Pathways and path lengths are supplied in Table 3. Trimer systems exhibited similar shifts in suboptimal paths.

**Table 3:** Suboptimal paths

C374 suboptimal path	PD	CD	PT	CT
	Connecting residues			
A7	1. R372 2. I369 3. E361 4. T358 5. A131 6. P102	1. I357 2. A131 3. P102	1. R372 2. I369 3. E361 4. T358 5. A131 6. P102	1. V370 2. I357 3. A131 4. P102
S14	N/A	1. Y133 2. L104 3. V9 4. K18 5. L16	1. R372 2. S368 3. E117 4. N115 5. I76 6. I71	1. V370 2. E117 3. N115 4. I76 5. I71
H73	1. R372 2. I369 3. P367 4. E117 5. N115 6. I75	1. H371 2. E117 3. N115 4. I75	1. R372 2. S368 3. E117 4. N115 5. I75	1. V370 2. E117 3. N115 4. I75
H88	1. R372 2. I369 3. P367 4. E117 5. N115 6. W79 7. M82 8. I85	1. Y133 2. L104 3. C10 4. W86	1. R372 2. S368 3. E117 4. M119 5. W86	1. R116 2. K118 3. W79 4. M82 5. I85
E107	1. R372 2. V370 3. R116	1. V134	1. R372 2. S368 3. E117 4. K113 5. N111	1. V134
N111	1. R372 2. I369 3. P367 4. E117 5. K113	1. H371 2. K113	1. R372 2. S368 3. E117 4. K113	1. V370 2. E117 3. N115
Q137	1. R372 2. V370 3. R116 4. E107	1. Y133 2. A135	1. R372 2. S368 3. E117 4. A114 5. P112 6. L110 7. A108	1. R116 2. E107
H161	1. R372 2. V370 3. R116 4. E107 5. I136 6. A138 7. V163	1. V134 2. E107 3. P109	1. R372 2. S368 3. E117 4. A114 5. P112 6. L110 7. R177	1. V134 2. E107 3. I136 4. A138 5. V163
R177	1. R372 2. V370 3. R116 4. E107 5. I136 6. A138 7. V163 8. H161	1. V134 2. E107 3. P109 4. H161	1. R372 2. S368 3. E117 4. A114 5. P112 6. L110	1. V134 2. E107 3. I136 4. A138 5. V163 6. H161
M305	1. R372 2. I369 3. E361 4. T358 5. A131 6. L8 7. G20 8. W340 9. S338 10. R335	1. Y133 2. A135 3. Q137 4. D154 5. G301	1. R372 2. S368 3. E117 4. A114 5. P112 6. L110 7. R177 8. T160 9. S155 10. G302	1. V134 2. E107 3. Q137 4. D154 5. G301

N/A, not available.

The shortest pathways connecting residue C374 with other key regions of actin’s structure were identified *via* dynamic network analysis. Listed are the residues to which pathways were identified from C374 (A7, S14, H73, H88, E107, N111, Q137, H161, R177, and M305). For each system, the intermediate connecting residues for the shortest path are listed, starting from C374.

### Allosteric conformational changes

Analyses so far have suggested that differences between PBM and control structures are concentrated around the nucleotide cleft. We therefore focused subsequent analyses on residues connecting the C terminus to the nucleotide cleft, as well as those that regulate nucleotide hydrolysis and release dynamics.

**Figure 7:**
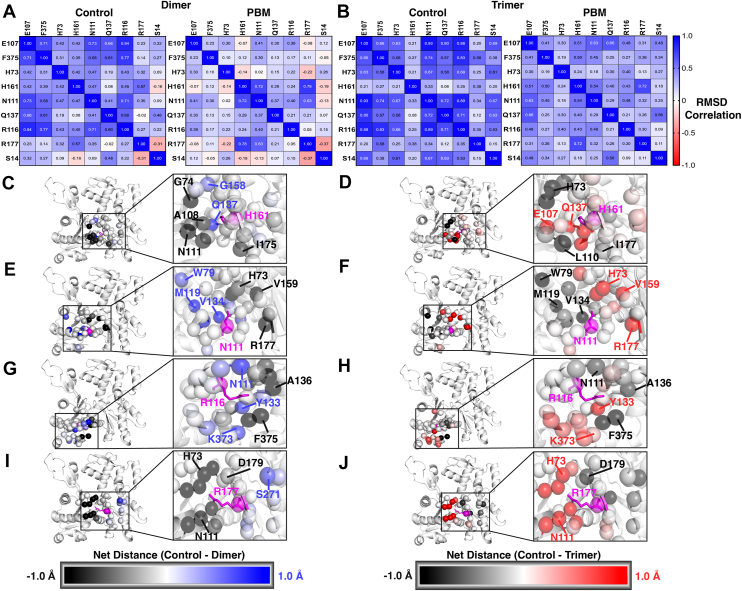
**Allosteric changes.** Structural shifts between connected residues were measured. RMSD, a measure of the average difference between two conformations, was calculated for several residues (E107, F374, H73, H161, N111, Q137, R116, R177, and S14) at each time point relative to their starting conformation (*t* = 0 ns). A correlation analysis was performed for (*A*) protomers CD3 and PD3 and (*B*) protomers CT4 and PT4 to determine if residues change conformations (*i.e.* change in RMSD) at the same time points, indicative of connected motions. A heat map of Pearson's coefficients was plotted, with *dark blue* representing values closer to 1 (strong positive correlation) and *red* representing values closer to −1 (strong negative correlation). *C*–*J*, average residue–residue distances were measured between all residues in the structure and target residues, H161, N111, R116, and R177. For all residues within 10A of the target residues, the difference in residue–target residue distance (control – PBM) was calculated and projected onto the structure, with residue alpha carbons represented as *spheres*, protein backbone represented as a *cartoon*, and target residues are colored *magenta* and represented as *sticks*. Negative values (PBM > control), colored *black*, indicate that residues reside closer to the target in control structures. Positive values (control > PBM), colored *blue* (dimer) and *red* (trimer), indicate that residues reside closer to the target in PBM structures. CD, control dimer; PD, PBM-dimer.

A correlation analysis was conducted to determine if a range of residues demonstrates similar RMSD shifts at the same time points (Fig. 7, *A* and *B*), indicative of coordinated shifts. Both PBM structures displayed a broad decrease in correlation, apart from increased correlations between H161–N111, H161–R177, and N111–R177 in the PD (Fig. 7*A*), as well as E107–H161, H161–N111, and H161–R177 in the PT (Fig. 7*B*).

Finally, the average distances between residues H161, N111, R116, and R177 and all other residues within 10A were measured, and the difference in residue–residue distances was projected onto the structure (Fig. 7, *C*–*J*). In both systems, Q137–H161, R116–Y133, and R116–K373 distances were reduced in the PBM structures. Relative to control, R177 and H73 were further away from N111 in PD and closer to N111 in PT, whereas R116 was further away from F375 and C374.

### Nucleotide cleft architecture and the P_i_ release gate

**Figure 8:**
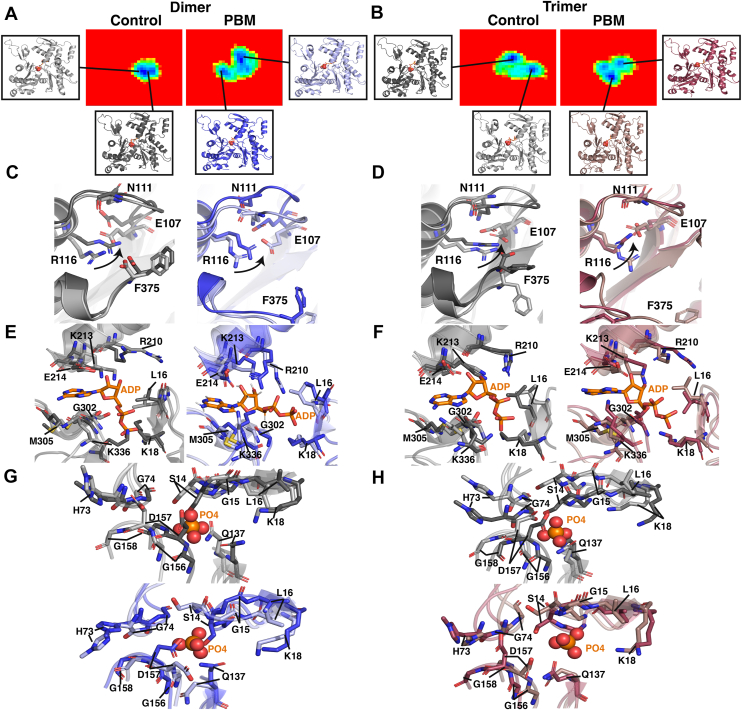
**Changes to nucleotide cleft architecture.** Structural shifts in the nucleotide-binding cleft were studied *via* structural superpositions of energetically favorable conformations. Structures from distinct minima in the Gibbs free energy landscapes (FELs), which represent the most favorable conformations sampled, were isolated for (*A*) the CD3 protomer (colored *light gray* and *dark gray*) and PD3 protomer (colored *light blue* and *dark blue*), as well as (*B*) the CT4 protomer (colored *light gray* and *dark gray*) and PT4 protomer (colored *light red* and *dark red*). *C* and *D*, superpositions of the CD3 (*light gray*, *dark gray*), PD3 (*light blue*, *dark blue*), CT4 (*light gray*, *dark gray*), and PT4 (*light red*, *dark red*) structures associated with each minimum show structural changes at the C terminus. In all systems, minima are defined by the position of R116, which can rotate inward toward E107 and N111, darker colored protomers representing inward shifts of R116. *E* and *F*, superpositions of the CD3 (*light gray*, *dark gray*), PD3 (*light blue*, *dark blue*), CT4 (*light gray*, *dark gray*), and PT4 (*light red*, *dark red*) structures associated with each minimum in the vicinity of bound ADP (*orange*) in the nucleotide-binding cleft. Shifts between minima at residues L16, K18, R210, K213, E214, G302, M305, and K336 are highlighted. *G* and *H*, superpositions of the CD3 (*light gray*, *dark gray*), PD3 (*light blue*, *dark blue*), CT4 (*light gray*, *dark gray*), and PT4 (*light red*, *dark red*) structures associated with each minimum in the vicinity of bound inorganic phosphate (P_i_, *orange*) in the nucleotide-binding cleft. Shifts between minima at residues S14, G15, L16, K18, H73, G74, Q137, G156, D157, and G158 are highlighted. CD, control dimer; PD, PBM-dimer; PT, PBM-trimer.

As nucleotide-binding cleft architecture might be altered by C-terminal shifts at the C terminus, structural superpositions were examined in the vicinity of ADP and P_i_ for structures corresponding to Gibb’s FEL minima (Fig. 8, *A* and *B*). Conformational differences are seen in the D-loop, H-plug, vicinity of the nucleotide cleft, and C terminus. In PBM clusters, F375 exhibits large shifts because of the PBM crosslink pulling down on C374, disrupting the F375–R116 interaction, which causes the side chain of R116 to oscillate between the C terminus and E107. Rotations toward E107 are associated with shifts in the N111 loop (residues 107–112) as well as the sensor loop (residues 72–77), residues 155 to 160, and the S14 loop (residues 12–16). Control systems exhibited smaller shifts, with clusters separated by F375 rotations that cause R116 to shift toward E107 and N111, affecting residues surrounding the nucleotide cleft.

Furthermore, differences in solvent-accessible surface area (SASA) were measured and projected onto the structure (Fig. 3, *A* and *B*). Several residues within the cleft exhibited variable SASA, including K336, E107, and M305, as well as N111 and R177 in the vicinity.

**Figure 9:**
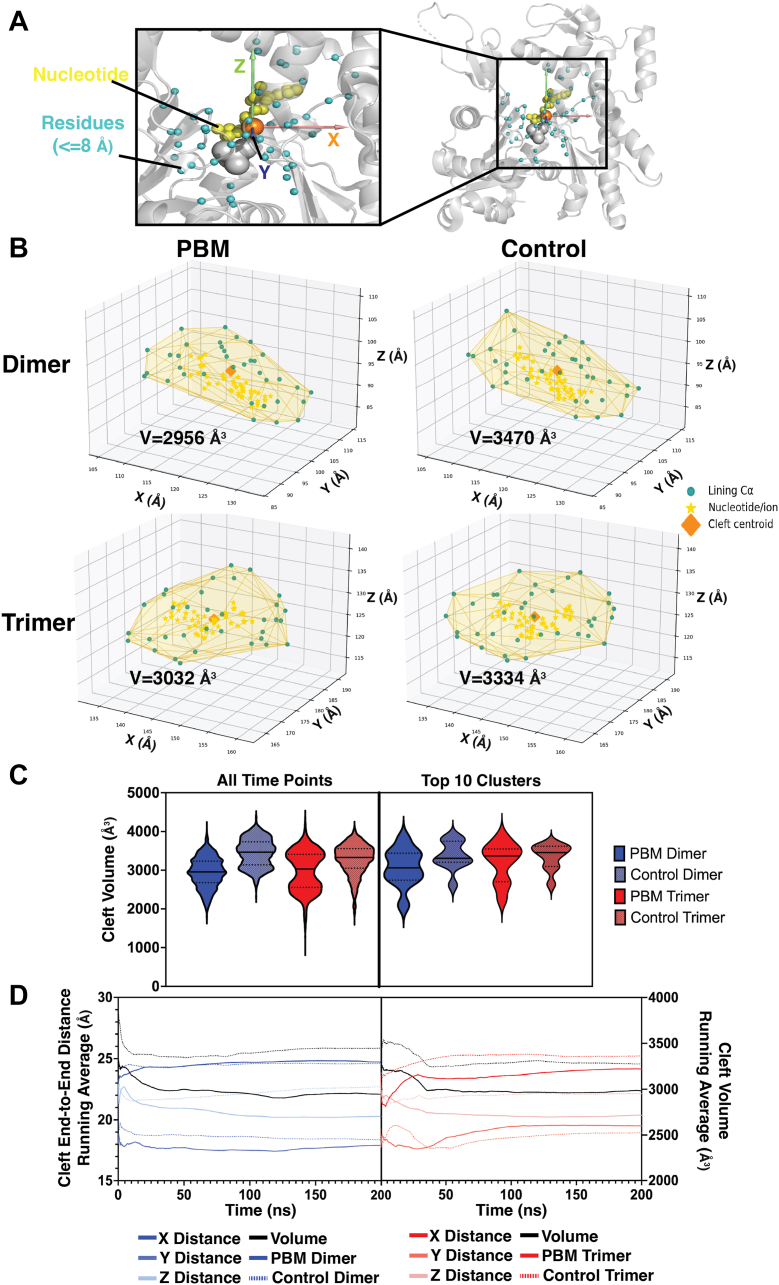
**Changes to nucleotide cleft volume.** The volume of the nucleotide cleft was measured. *A*, to define the nucleotide cleft, all residues within 8 Å (*cyan*) of ligands within the cleft (*yellow*) were selected at each time point, and their coordinates were extracted. A convex hull connecting these residues was generated, and the volume of this hull was calculated. *B*, the median convex hull for each system was plotted, showing the positions of alpha-carbons for all selected residues (*cyan*), the centroid of these residues (*orange*), and the position of nucleotides at that time point (*yellow*). *C*, the nucleotide cleft volume at each time point, as well as the top 10 structural clusters, was plotted for each system. Mean volumes are identified by *solid lines* within the violin plots, whereas quartiles are represented by *dotted lines. D*, the running average of the end-to-end distance (Å) along the *X*- (*dark blue*, *dark red*), *Y*- (*medium blue*, *medium red*), and *Z*-axes (*light blue*, *light red*) was calculated and plotted for all time points for dimer (*blue*) and trimer (*red*) systems. PBM systems are represented by *solid lines*, whereas control systems are represented by *dashed lines*. The running average cleft volume is plotted (colored *black*) on the right *Y*-axis. PBM, *N*,*N*′-*para*-phenylenebismaleimide.

The volume of the nucleotide cleft was measured at each time point as well as for the top 10 structural clusters (Fig. 9). To estimate cleft volume, all residues within 8 Å of ligands within the cleft were selected for each time point, from which a convex hull was constructed, and the volume was calculated. The median convex hulls for PBM systems were considerably smaller than their control counterparts (Fig. 9*B*), and PBM systems exhibited lower average volumes over time (Fig. 9*C*). The end-to-end distances in *X*, *Y*, and *Z* directions were measured, indicating that differences in cleft volume arise from decreased *Z*-axis distances in PBM systems (Fig. 9*D*).

Finally, changes in dihedral angles were measured (Fig. 3, C–E). Q137 saw shifts in X1 and X2 dihedral angles relative to control structures, whereas S14 displayed changes in X1, phi, and psi angles in both PBM structures.

**Figure 10:**
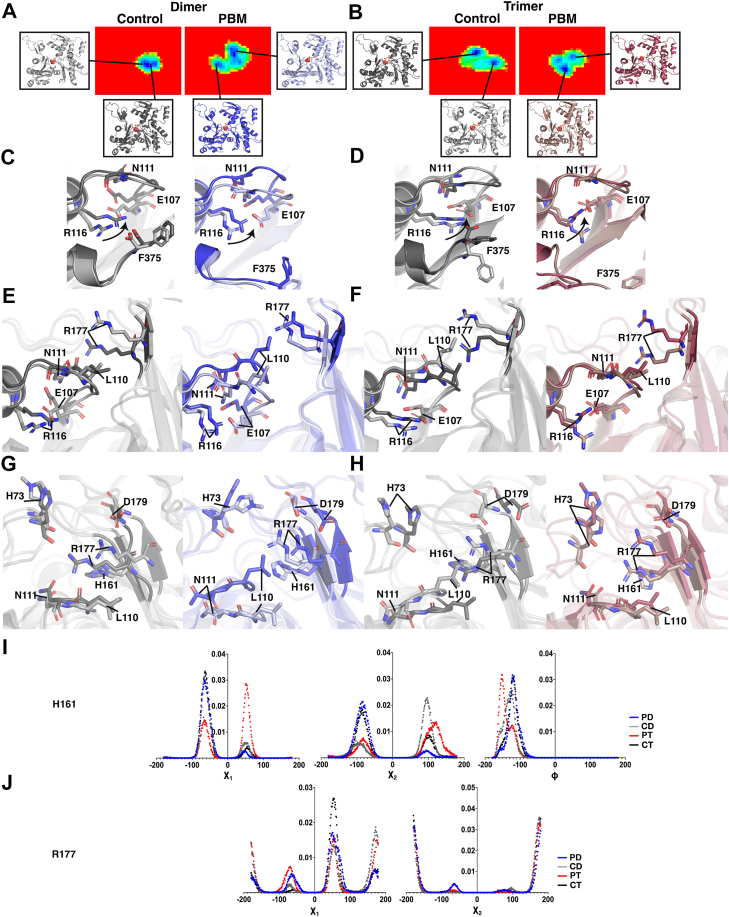
**Changes to the phosphate release gate.** Structural shifts in the vicinity of the phosphate release gate were studied *via* structural superpositions of energetically favorable conformations. *A* and *B*, structures were isolated from distinct minima in the Gibbs free energy landscapes (FELs), which represent the most favorable conformations sampled. The control-dimer structures were compared for the CD3 protomer (light gray, dark gray), PBM-dimer structures were compared for the PD3 protomer (*light blue*, *dark blue*), control-trimer structures were compared for the CT4 protomer (*light gray*, *dark gray*), and PBM-trimer structures were compared for the PT4 protomer (*light red*, *dark red*). *C* and *D*, structural superpositions of isolated structures in the vicinity of the C terminus show rotations of R116 inward toward N111 and E107 in the darker colored structures for all systems. *E* and *F*, the phosphate release gate (P_i_-gate) is formed by shifting hydrogen bonds between R177 and residues N111 or L110. Rotations of R116 toward N111 can affect the loop containing residues 107 to 112, impacting stability and formation of the P_i_-gate. Shifts in this region between distinct minima of the FELs are compared *via* structural superpositions. *G* and *H*, opening of the P_i_-gate is regulated by H161, which breaks R177’s hydrogen bonds and allows it to shift into the open position, which is stabilized by D179. Methylated H73 forms part of R177’s hydrogen bond network that stabilizes the P_i_-gate. Shifts in these residues are compared between energetically favorable conformations to observe changes in P_i_-gate formation. *I*, histograms of H161 dihedral angles (Χ_1_, Χ_2_, and Φ) are shown for PD (*blue*), CD (*gray*), PT (*red*), and *CT* (*black*) systems to identify variable rotameric conformations that might impact the P_i_-gate. *J*, histograms of R177 dihedral angles (Χ_1_, Χ_2_) are shown for PD (*blue*), CD (*gray*), PT (*red*), and CT (*black*) systems to identify variable rotameric conformations indicating changes to the P_i_-gate. CD, control dimer; CT, control trimer; PBM, *N*,*N*′-*para*-phenylenebismaleimide; PD, PBM-dimer; PT, PBM-trimer.

Structural changes might also affect P_i_ release dynamics. Residues N111–R177 form a gate regulating P_i_ release (21), dictated by rotameric shifts in H161 (22). In all systems, rotations of R116 toward E107 and N111 (Fig. 10, *C* and *D*) alter the conformation of the N111 loop, impacting the distance between R177 and N111 (Fig. 10, *E*–*H*). Shifts in R177 and H161 dihedral angles are also observed in PBM systems, relative to control (Fig. 10, *I* and *J*).

### Nucleotide dynamics

**Figure 11:**
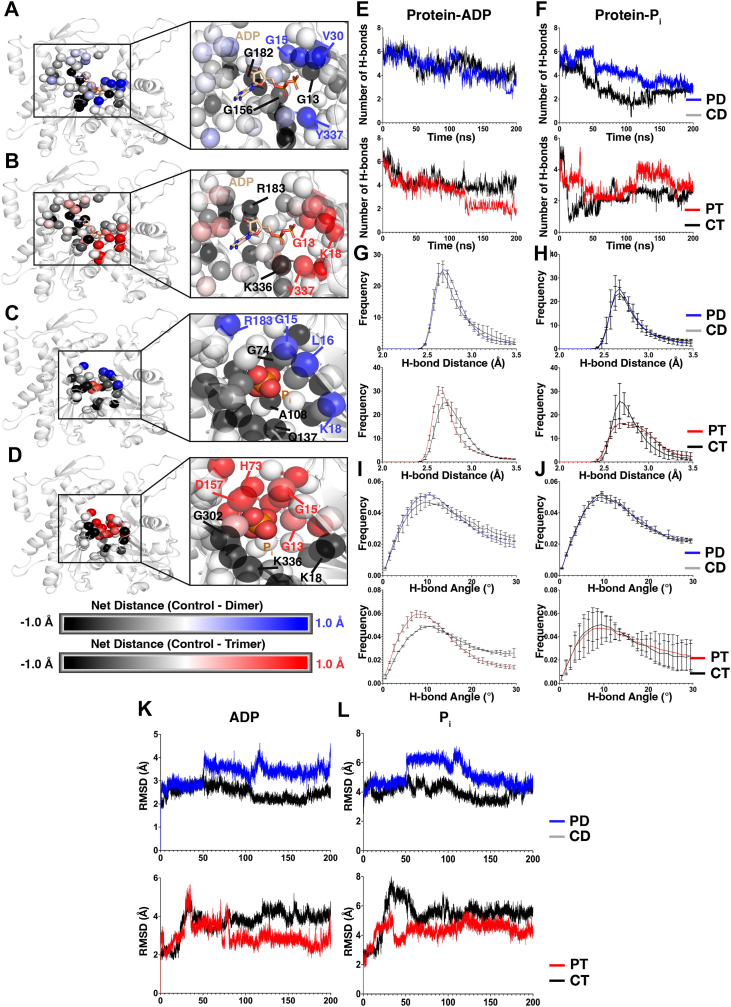
**Changes to nucleotide dynamics.***A* and *B*, average distances from ADP (*orange*) to all residues within 10A were calculated for each system. Differences between control and PBM systems (control – PBM) were calculated and projected onto the structure. Negative values (PBM > control, colored *black*) represent residues in control systems that are closer on average to ADP throughout the simulation, relative to PBM. Positive values (control > PBM, colored *blue* for dimer, colored red for trimer) represent residues in PBM systems that are closer on average to ADP throughout the simulation, relative to control. Residue alpha carbons are represented by *spheres*. *C* and *D*, average distances from P_i_ (*orange*) to all residues within 10A were calculated for each system. Differences between control and PBM systems (control – PBM) were calculated and projected onto the structure. Negative values (PBM > control, colored *black*) represent residues in control systems that are closer on average to P_i_ throughout the simulation, relative to PBM. Positive values (control > PBM, colored *blue* for dimer, colored *red* for trimer) represent residues in PBM systems that are closer on average to P_i_ throughout the simulation, relative to control. Residue alpha carbons are represented by *spheres*. *E*, average number of protein–ADP hydrogen bonds over time for PD (*blue*), CD (*gray*), PT (*red*), and CT (*black*) systems. *F*, average number of protein-P_i_ hydrogen bonds over time for PD (*blue*), CD (*gray*), PT (*red*), and CT (*black*) systems. *G*, histograms of protein–ADP hydrogen bond distances for PD (*blue*), CD (*gray*), PT (*red*), and CT (*black*) systems. Error bars represent standard deviation. *H*, histograms of protein-P_i_ hydrogen bond distances for PD (*blue*), CD (*gray*), PT (*red*), and CT (*black*) systems. Error bars represent standard deviation. *I*, histograms of protein–ADP hydrogen bond angles for PD (*blue*), CD (*gray*), PT (*red*), and CT (*black*) systems. Error bars represent standard deviation. *J*, histograms of protein-P_i_ hydrogen bond angles for PD (*blue*), CD (*gray*), PT (*red*), and CT (*black*) systems. Error bars represent standard deviation. *K*, average RMSD values over time for ADP in PD (*blue*), CD (*gray*), PT (*red*), and CT (*black*) systems. *L*, average RMSD values over time for P_i_ in PD (*blue*), CD (*gray*), PT (*red*), and CT (*black*) systems. CD, control dimer; CT, control trimer; PBM, *N*,*N*′-*para*-phenylenebismaleimide; PD, PBM-dimer; PT, PBM-trimer.

All structural analyses suggest that nucleotide cleft architecture, and therefore nucleotide dynamics, might be altered in PBM structures. Net distance between ADP and P_i_ and nucleotide cleft residues was calculated (Fig. 11, *A*–*D*). In PBM systems, ADP existed closer to the corner of the nucleotide cleft containing residues Y337 and V30, whereas P_i_ existed closer to the top of the cleft around residues R183 and G15. In control systems, ADP resided closer to the corner of the nucleotide cleft containing residues G182–R183, whereas P_i_ resided closer to the bottom of the cleft containing residues G302, K336, and Q137.

Hydrogen bond properties were also calculated (Fig. 11, *E*–*J*). No difference in the number of ADP:protein bonds was observed in dimer systems, whereas PT contains fewer ADP:protein hydrogen bonds (Fig. 11*E*). For P_i_, both PBM systems displayed more hydrogen bonds for much of the simulation before converging with the control structures (Fig. 11*F*). Histograms of hydrogen bond distances (Fig. 11, *G* and *H*) and angles (Fig. 11, *I* and *J*) were also plotted, showing shifts in the bond distances for ADP and P_i_ in PT as well as shifts in the ADP hydrogen bond angles in both PBM systems.

Ligand RMSD values were plotted over the course of the simulations (Fig. 11, *K* and *L*), with ADP and Pi displaying greater RMSD in the PD structures relative to control and reduced RMSD in PT relative to control.

## Discussion

Substantial evidence indicates that actin’s C terminus plays an active role in actin structural changes: it exists at the main site for ABP binding in both G- and F-actin (2), it forms the interprotomer interface, which guides actin filament properties (8, 23), it is inherently flexible (16, 24) and appears to shift conformations upon ABP binding (17, 25, 26), and conformational changes at the C terminus have been connected to changes in distant regions (27). For example, the binding of many ABPs to the interprotomer interface in F-actin or the C terminus in G-actin is dependent on actin’s nucleotide state, with recent EM structures identifying a clear pathway from Q137 in the nucleotide cleft to F375 at the C terminus (7). It was therefore suggested that actin’s C terminus responds to nucleotide dynamics, altering the architecture of the interprotomer interface, regulating ABP binding through direct conformational detection or alteration of filament mechanical properties. While actin’s C terminus appears to respond to changes in the nucleotide state, we have previously proposed that such communication flows in both directions (18), with actin’s C terminus acting as a nexus point for conformational changes within cellular interactions. In this study, we examined this hypothesis by observing the impacts of altering C-terminal properties with PBM crosslinks.

### PBM crosslinks do not substantially alter filamentous structure

Previous *in vitro* studies utilizing PBM–actin (8, 15, 28) were conducted prior to the development of high-resolution structural models (8). As such, structural mechanisms driving *in vitro* observations could not be elucidated at that time. It was observed, however, that ABPs such as myosin could effectively bind to PBM–actin filaments (15). While there was functional inhibition of myosin, these results suggested that PBM-crosslinks did not substantially alter F-actin structures, as large-scale rearrangements would likely cause filament instabilities and interfere with ABP binding.

Our simulations align with these results, as we observed that structures were overall similar with highly localized conformational shifts. RMSD and radius of gyration were similar for all systems, suggesting similar levels of protomer-wide shifts (Fig. 2). RMSF demonstrated similar flexibility in PBM and control systems (Fig. 2, *D* and *E*), with changes concentrated at the C terminus, D-loop, nucleotide cleft, and the top of the bumper helix, whereas RMSD values demonstrated similar localization of structural changes (Fig. 4, *A* and *B*). Finally, average domain rotation is similar between control and PBM structures (Fig. S2).

As differences between PBM and control systems were heavily concentrated between the C terminus and the nucleotide-binding cleft, we next examined changes to known communication pathways between these regions.

### C-terminal shifts affect internal communication networks

We utilized PBM-induced changes at the C terminus to analyze critical communication pathways and identify regions responding to C-terminal shifts. While overall protomer conformations are similar between PBM and control systems, structures isolated from minima of Gibbs FELs show that the status of F375–R116 interactions and positioning of R116 are differentiating factors between energetically favorable states (Fig. 8, *C* and *D*). We therefore explored the impacts of disrupting F375–R116 interactions to understand its role in C-terminal communication.

Actin’s C terminus is connected to the D-loop within the protomer (27, 29). Previously, we proposed that C-terminal shifts pass through the pathogenic helix (residues 112–126) and a helix containing tryptophan residues (referred to here as W-helix, residues 78–91) to SD2 and the D-loop (18). The loss of F375–R116 connections should therefore impact communication through these structural elements. Dynamic network analyses (Fig. 5, *A* and *B*) indicated variable levels of connectivity within SD1 and fewer connections at the interface of SD 1 and 2. Suboptimal paths, a measure of the shortest route between two residues, indicated that communication between C374 and H88 (near SD2) involves less efficient pathways in PBM systems (Fig. 6, *B* and *D* and Table 3). Further, RMSD indicates differences between PBM and control systems at C374–F375, in the pathogenic helix near residue N111, the sensor loop (residues 72–77), T-helix, and the base of SD2 (Fig. 4, *A* and *B*). Taken together, these results support our previously proposed SD1−SD2 communication pathway. We conclude that, because of overall conformational similarities, impaired communication throughout SD1 likely stems from altered F375–R116 interactions. C-terminal positioning might therefore mediate overall allosteric communication through the F375–R116 interaction.

Recent high-resolution structures identified a clear pathway from the nucleotide-binding cleft to the C terminus through SD1 (7). As SD1–SD2 communication is impaired, we speculated that C-terminal–nucleotide communication would be similarly impacted. Dynamic network analyses indicated a loss of critical nodes through the nucleotide cleft in PBM systems (Fig. 5, *A* and *B*), whereas control systems also contained more unique connections centered around the nucleotide cleft (Fig. 5, *C* and *D*). Internal pathways critical to the broader network shift from the nucleotide cleft shifting to the periphery in PBM systems (Fig. 6, *A* and *C*), whereas suboptimal paths demonstrated that PBM systems rely on longer and less efficient communication pathways (Fig. 6, *B* and *D*). The C terminus and nucleotide cleft are connected through residues R116, E107, and Q137, whereas PBM systems are instead connected along the length of the N111 loop (Table 3). In addition, a pathway from C374 to M305 travels directly through the nucleotide-binding cleft in control systems compared with the Pi gate (R177) and SD3 in PBM. Finally, RMSD (Fig. 4, *A* and *B*), PCA analyses (Fig. 4, *C* and *D*), structural superpositions (Fig. 10), and residue–residue interactions (Fig. 7) all suggest that the largest structural changes occur at residues C374/F375, R116, the N111 loop, and the neighboring vicinity, including the sensor loop and R177. Substantial evidence therefore suggests that communication involving the C terminus and nucleotide cleft, which is critical for overall actin communication and function, is impaired by altering actin’s C terminus.

In summary, while the C terminus remains connected to distant structural elements, C-terminal shifts that alter F375–R116 interactions cause localized conformational changes around the nucleotide cleft and impair communication throughout the protomer. It is known that changes at the nucleotide cleft affect C-terminal conformation, but our and previous *in vitro* data suggest that C-terminal positioning can mediate distant conformational shifts through the same communication pathways.

### Actin’s C terminus influences nucleotide cleft architecture

If C terminus–nucleotide cleft communication is indeed bidirectional, we expect to see altered nucleotide cleft architecture and nucleotide dynamics because of C-terminal shifts.

Within the nucleotide cleft, Q137 participates in ATP binding and hydrolysis (21, 30) whereas H161 likely triggers Pi release by regulating the N111–R177 Pi gate (21). To understand how nucleotide cleft architecture shifts in response to the C terminus, we measured the correlation of residue movements throughout the simulation. We observed a broad reduction in residue correlations in PBM systems (Fig. 7, *A* and *B*), in line with impaired communication from network analyses. In both PBM structures, however, the exception to reduced correlations involves residue H161. Analyses of dihedral angles also indicate rotameric differences between H161, R177, and Q137 in PBM systems (Figs. 10, *I* and *J* and S3*C*). Furthermore, RMSF values indicate differences in flexibility around the nucleotide cleft (Fig. 2, *D* and *E*), H161–Q137 and N111–R177 distances are altered (Fig. 7, *C*–*J*), whereas PCA demonstrates an increased range of motion around the N111 loop, residue 177, and an opening of the back of the nucleotide cleft (Fig. 4, *C* and *D*). It therefore appears that PBM-induced changes at the C terminus lead to altered nucleotide cleft architecture, in agreement with previously observed rotations of SD1, leading to an opening of the nucleotide cleft (31).

To better understand changes involving actin’s nucleotide, we isolated structures associated with FEL energy minima and analyzed them *via* structural superpositions (Figs. 8 and 10). In PBM systems, the loss of stabilizing F375–R116 interaction allows R116 to freely rotate, associating with other C-terminal residues, such as K373, or internal residues, such as E107, N111, and Y133 (Fig. 7, *G* and *H*). Rotations of R116 toward internal residues appear to be connected to motions of the N111 loop and nearby elements, such as the sensor loop, S14 loop, and the stretch of residues containing R177, which hinges outward toward the H-plug. We also saw variable RMSF and RMSD in H73 and R177 (Figs. 2, *D* and *E* and 4, *A* and *B*), increased ranges of motion around N111 loop and R177 (Fig. 4, *C* and *D*), rotameric shifts in H161 and R177 (Fig. 10, *I* and *J*), as well as variable residue–residue distances and correlations involving H161, R177, and N111 (Fig. 7). Within the nucleotide cleft, rotameric shifts are seen in S14 and Q137 (Fig. S3, C and E), and residues throughout the cleft exhibit variable SASA (Fig. 3, *A* and *B*). The result of altered cleft architecture appears to be reduced nucleotide cleft volume (Fig. 9) in PBM systems because of reduced end-to-end distances along the *Z*-axis resulting from either upward shifts in the N111 loop or altered SD2–4 interactions because of shifts in the D-loop. Furthermore, structural superpositions show that ADP and P_i_ binding residues exhibit shifts (Fig. 8), leading to variable residue–ADP and residue–P_i_ distances (Fig. 11, *A*–*D*), which change hydrogen bond distances (Fig. 11, *G* and *H*), angles (Fig. 11, *I* and *J*), and affect nucleotide stability (Fig. 11, *K* and *L*). Finally, structural superpositions around the P_i_ gate (Fig. 10) suggest that changes in the F375–R116 interaction impact N111 loop–R177 interactions. Our data therefore suggest that a conformational shift in the C terminus can affect the position of R116, impacting residues associated with nucleotide hydrolysis (Q137) or initiation of P_i_ release (H161) as well as communication throughout SD1, likely altering nucleotide hydrolysis and release dynamics.

Whether P_i_ release involves a pore between S14 and G74 (30, 32, 33) or H161 opening the N111–R177 gate (21, 22, 34, 35), our results indicate that C-terminal changes impact the residues regulating nucleotide binding, hydrolysis, and release while causing changes to known communication pathways. It is therefore possible that C-terminal shifts drive the regulation of actin’s functional state through allosteric means.

### The functional implications of C-terminal shifts

Our results suggest a direct connection between actin’s C terminus and nucleotide dynamics, where C-terminal shifts influence nucleotide cleft architecture through allosteric pathways. Past studies have shown that modification of C374 affect ATPase activity (9), whereas C-terminal cleavage, mutagenesis, and the conjugation of fluorescent probes affects ATP hydrolysis (27), affinity (19, 27), and rate of exchange (19). Moreover, the stabilization of actin’s C terminus with phalloidin (36) can delay Pi release (33) and restore ATPase activity in truncated actin (9). Our results are therefore supported by a wealth of *in vitro* studies demonstrating a connection between the actin’s C terminus and nucleotide dynamics, with significant functional implications.

The C terminus is part of the main binding site for ABPs in G-actin. Many ABPs bind to the C terminus in a nucleotide-dependent manner (37, 38) whereas ABPs, such as profilin, bind to the C terminus and alter nucleotide cleft architecture (39, 40, 41) to affect nucleotide affinity and exchange (42). Disruption of the C-terminal–nucleotide cleft communication, as we observed, will likely impact ABP binding affinity and functions.

Actin’s C terminus also regulates ABP binding and function in F-actin, as C-terminal cleavage has been shown to affect filament properties (43) and reduce ABP binding (44, 45) whereas PBM crosslinks result in an uncoupling of actomyosin ATPase activity and force generation (15, 28). Our data suggest three possible mechanisms of actomyosin inhibition by PBM crosslinking.

Myosin binds to the interprotomer interface formed between the C terminus and D-loop of neighboring intrastrand protomers as well as the H-plug of their cross-strand neighbor. As the C terminus is pulled out of position by PBM crosslinks and structural changes are seen in the D-loop (Fig. 3) and H-plug, the makeup of this interface could be altered and directly affect myosin attachment–detachment kinetics. As overall structural changes are small, differences at the interprotomer interface could explain why myosin binds to PBM filaments normally while force generation is impacted. Alternatively, the interprotomer interface dictates F-actin mechanical properties (31, 46, 47) which might be sensed by ABPs (23). As myosin depends on filament flexibility (48), altered mechanical properties because of changes at the interprotomer interface could reduce force generation. As myosin is affected by actin’s nucleotide state (49) and a direct pathway between the nucleotide cleft and the C terminus has been identified (7), myosin kinetics likely depend on actin communication networks. Furthermore, myosin binding induces a shift in the C terminus toward SD1 (16, 50, 51) and it has been shown that myosin binding is cooperative (52, 53, 54), affecting actin filaments up to 25 protomers away from the binding site (48, 55) through long-range propagation (56, 57). Therefore, C-terminal changes induced by PBM crosslinks likely prohibit the necessary shifts upon myosin binding that facilitate long-range cooperative binding.

All possible mechanisms of actomyosin inhibition presented suggest that actin’s C terminus regulates the conformations facilitating myosin binding and activity, with similar dynamics regulating the binding and functions of other ABPs. Due to the location of actin’s C terminus at the main ABP-binding site, known C-terminal shifts upon ABP binding, and actin’s connections to distant structural elements, it is possible that actin’s C terminus acts as a nexus for structural changes, facilitating transitions that enable actin’s functions within cellular processes.

### Future directions

The focus of this study was to use PBM as a mechanism of modifying actin’s C terminus for measuring internal responses, potentially connecting such responses to previous functional data. Our results support the suggestion that C-terminal conformational shifts are connected to actin’s nucleotide cleft architecture, though further studies are needed to reveal the exact mechanisms driving these changes. While the work presented here improves our understanding of actin’s role in cellular processes, the role of actin’s C terminus in structural changes is just one piece of the puzzle within actin dynamics. Further studies utilizing high-resolution EM in conjunction with MD simulations can identify specific pathways driving or responding to C-terminal changes within functional shifts. For example, no EM structure of PBM–actin filaments exists. While the starting structures for our simulations represent reasonable estimations of PBM actin, resolving EM structures of PBM–actin filaments can confirm our results. Furthermore, structural changes propagate along the length of actin filaments, altering filament bending and mechanical properties. While such computations are beyond the scope of this study, coarse-grained MD simulations can model long-range actin dynamics influencing ABP binding and functions. Such follow-up studies can strengthen our understanding of how C-terminal flexibility induces long-range changes in actin filaments and dynamic shifts between functional states.

A C-terminal induced shift in actin’s functional state likely involves the nucleotide-binding cleft, as nucleotide hydrolysis and phosphate release dynamics regulate actin’s functions. In this study, we extrapolated changes to nucleotide dynamics from predicted structural changes, but MD simulations are not conducted on the time scales needed to model phosphate release. This analysis can be achieved through subsequent metadynamics studies, where bias potentials improve conformational sampling by flattening energy barriers to model processes on larger time scales.

Finally, our simulation results identified common regions experiencing conformational shifts in PD and PT, suggesting direct connections to actin’s C terminus. Though the structural elements exhibiting shifts were common to both systems, PD and PT exhibited some differences that can be explained by variable interprotomer dynamics, which are unavoidable in F-actin systems. Interprotomer interactions might therefore exacerbate structural changes observed in PBM filaments. MD simulations of monomeric G-actin can identify intraprotomer structural changes by removing the influence of interprotomer knock-on effects. Selected mutagenesis within SD1, such as alanine scanning or targeted deletions, can be utilized to alter C-terminal conformation to identify how changes propagate within the protomer. Furthermore, simulations of additional crosslinkers of varying lengths and orientations could be used to modify the C terminus in similar but distinct ways, providing further data on the impacts of altering C-terminal orientation and interactions.

## Conclusions

Actin is essential for cellular processes through interactions with ABPs. Many of actin’s interactions involve its highly flexible C terminus, which undergoes conformational shifts when many ABPs bind. Actin’s C terminus has also been connected to distant structural elements through internal communication networks, such as the nucleotide-binding cleft for nucleotide-dependent ABP binding. Despite knowledge of C-terminal importance, however, resolution constraints have hampered studies seeking details of C-terminal regulation.

Here, we conducted *in silico* modeling of actin with C termini modified by PBM crosslinks. We examined how C-terminal conformation regulates internal communication networks and functional changes within actin. We determined that changes to stabilizing R116–F375 interactions disrupt protomer-wide communication networks, inducing localized conformational changes in key areas, including the nucleotide-binding cleft. Residues guiding nucleotide binding, hydrolysis, and release exhibit conformational changes that appear to alter nucleotide binding dynamics. Our results support past *in vitro* studies suggesting that C-terminal conformational changes play a critical role within actin’s interactions and structural changes for cellular functions.

Past structural and functional studies, in conjunction with the results presented here, have led us to propose that actin’s C terminus acts as a nexus point for regulating actin structural changes through internal communication pathways. It has been shown that a wide range of ABPs bind to the C terminus and induce a conformational shift. C-terminal stability and structural integrity maintain filament properties that ABPs rely on, actin’s C terminus is connected to distant structural elements in both G- and F-actin, and actin’s C terminus responds to nucleotide changes for nucleotide-dependent ABP binding. Furthermore, actin’s C terminus is located at the main ABP-binding site in both G- and F-actin, is highly conserved, and has wide-ranging allosteric connections that are thought to apply selective pressure on actin (58). As our results connect C-terminal conformation to the maintenance of communication networks and nucleotide cleft architecture, it is possible that C-terminal shifts act as a “switch,” utilizing allosteric communication to facilitate shifts between functional states. Within this model, actin’s C terminus can shift in response to stimuli such as nucleotide hydrolysis or ABP binding, inducing long-range structural changes to adopt a functional state that enables cooperative ABP binding or subsequent changes in the nucleotide cleft.

Actin plays a critical role in cellular processes, with actin’s C terminus potentially regulating actin’s structural changes within these processes. Continuing to understand how actin’s C terminus might regulate actin’s functions through allosteric mechanisms will therefore improve our understanding of fundamental processes for life on earth.

## Experimental procedures

### Computational models

To better understand the structural impacts of PBM crosslinks, two systems were examined: a six-protomer actin filament composed of three dimers (F-dimer) and a nine-protomer actin filament composed of three trimers (F-trimer). One protomer was isolated from PDB 8A2S (F-actin in the Mg^2+^ ADP–Pi state) to serve as a reference structure. MODELLER was used to generate structural models of skeletal actin from the reference 8A2S protomer, building residues missing from the 8A2S PDB. Ten potential models were then assessed using the MODELLER objective function and discrete optimized protein energy scores. Methylated histidine 73, Mg^2+^, ADP, and P_i_ were docked to the model using structural superpositions with the 8A2S reference protomer in PyMOL (The PyMOL Molecular Graphics System, version 2.0; Schrödinger, LLC).

Two and three copies of this model were then used to form a single dimer (chains 1 and 2) and trimer (chains 1–3), respectively, *via* structural superpositions to PDB 8A2S in PyMOL. A PDB for PBM was built using the CHARMM-GUI ligand reader (59, 60), which served to generate PBM parameters for implementation into the CHARMM36 forcefield (61, 62) was then docked in PyMOL to F-dimer residues C374 (chain 1) and K191 (chain 2), and F-trimer residues C374 (chains 1/2) and K191 (chains 2/3). F-dimer and F-trimer, comprising six and nine protomers, respectively, were then built *via* structural superpositions of the individual dimers and trimers to PDB 8A2S. PBM-free control filaments were also built, comprising the same number of protomers. In total, four F-actin systems were generated: PBM-F-dimer (PD), PBM-free F-dimer control (CD), PBM–F-trimer (PT), and PBM-free F-trimer control (CT), as shown in Figure 1.

Parameters for PBM were added to the CHARMM36 forcefield (61, 62) and the GROMACS (63) file specbond.dat was modified to add covalent linkages between PBM and the side chains of C374 and K191. Furthermore, CHARMM36 files were altered to recognize PBM-linked cysteine and lysine as protein residues by defining linkages between PBM maleimide carbons and cysteine’s/lysine’s side-chain sulfur and nitrogen, respectively. Residue-PBM dihedral parameters were generated *via* analogy using predefined residue–ligand linkages contained in the CHARMM36 forcefield. The CHARMM36 forcefield in GROMACS was utilized for all MD simulations through the Digital Research Alliance of Canada’s Graham computing cluster, located at the University of Waterloo. The TIP3P explicit water model (64) with Na^+^ and Cl^-^ counter ions was used to solvate the system with a neutral charge and 150 mM salt concentration. The protein was centered within a cubic box at a minimum distance of 10 Å from the box edge. Periodic boundary conditions were applied in all directions, and the GROMACS-recommended CHARMM36 parameters were used. The system was minimized with a steepest descent step size of 0.05 until the maximum force was less than 1000 kJ mol^−1^nm^−1^. The system was equilibrated at 310 K and 1 bar over 100 ps, before simulations were conducted over 200 ns in duplicate (for an overview of systems studied, see Tables 1 and 2) using the Berendsen thermostat and Parrinello–Rahman barostat (65) with a 2 fs timestep. Bond constraints utilized the LINCS algorithm (66), the particle-mesh Ewald method was used for long-range electrostatics, and short-range electrostatics and van der Waal’s forces were cutoff at 1.2 nm. Position restraints for maintaining filament symmetry were applied to the backbone of F-actin residues 105, 162, and 157 in the protomers at the top and bottom of the filament (chains 1+2+5+6 for F-dimer systems, chains 1+2+8+9 for F-trimer systems), leaving the central dimer (chains 3+4) and trimer (chains 4–6) free of restraints.

### Analysis of MD simulations

In-house scripts were used for all analyses. MD trajectories were first processed to account for periodic boundary jumps before individual protomers (chains 3+4 for F-dimer, chains 4–6 for F-trimer) were isolated for analysis. Protomers within the center of the filament (*i.e.*, chain 3 for F-dimer, chain 4 for F-trimer) were chosen as they maintain protein–protein contacts in all directions. Due to the constraints at the top and bottom of the filament, central protomers are therefore representative of protomers within a larger filament. RMSD, RMSF, radius of gyration, hydrogen bonds, and bond distances were calculated from the individual replicate trajectories.

To assess D-loop positions, custom Python scripts were crafted to isolate D-loop coordinates over time using MD analysis (67, 68), before plotting with Matplotlib (69). In each simulation frame, the centroids of each SD were determined by calculating the mean coordinates of all residues within each SD. Similar to methods used by Oda *et al*. (6) to calculate domain tilt, an axis system was defined where the *X*-axis was the vector connecting the centroids of SDs 1/3, and the *Z*-axis was perpendicular to the *X*-axis in the direction of SD 2. The angles of structural elements such as the D-loop could then be calculated within this axis system to assess changes in residue positions. For examining changes to the nucleotide cleft, a convex hull was generated within the axis system. All residues within 8 Å of all ligands were selected, and the volume of a convex hull connecting these residues was calculated with SciPy (70). Domain tilt was calculated by comparing the angle, relative to the *Z*-axis, of the vector connecting the centroids of SDs 1 to 2 to that of the vector connecting the centroids of SDs 3 to 4.

Combined trajectories were produced for each system (*i.e.*, the two 200 ns replicates for PD were combined into a single 400 ns trajectory). Combined trajectories were utilized for cluster analyses using the GROMOS algorithm (71) and a cutoff of 0.15 nm. Dynamic network analyses were conducted using the NetworkView plugin (72) in VMD (73). Trajectories containing only protein atoms were converted to .dcd format, whereas .psf files were generated from protein-only .pdb files. Alpha-carbons as node selections were used for network analyses, whereas the Girvan–Newman algorithm (74) identified communities of correlated residues using default cutoffs.

To identify dominant protein motions, PCA was conducted for the combined trajectories of chains 3 + 4 for F-dimer systems and chains 4 to 6 for F-trimer systems. The two largest vectors of motion for all protein atoms (*i.e.*, excluding all ligands, including PBM), referred to as PCs 1 and 2, respectively, were projected onto the structure of each chain to visualize extreme motions. To compare the conformational freedom within each system, the PD trajectory was combined with the six protomer CD trajectory for all protein alpha-carbons, whereas the PT trajectory was combined with the nine protomer CT trajectory for all protein alpha-carbons, resulting in two 800 ns combined trajectories (400 ns PD + 400 ns CD, 400 ns PT + 400 ns CT, alpha-carbons only). PCA was conducted on both 800 ns combined trajectories, identifying dominant motions common to control and PBM-crosslinked systems. The individual trajectories (*i.e.*, 400 ns PD, 400 ns CD, 400 ns PT, and 400 ns CT) were projected along the respective 800 ns eigenvectors, which were used to generate Gibbs FELs.

## Data availability

All data for this publication are presented in the article and supporting information. Excel files containing raw data are available upon request from John F. Dawson (jdawso01@uoguelph.ca).

## Supporting information

This article contains supporting information.

## Conflict of interest

The authors declare that they have no conflicts of interest with the contents of this article.
