# Supplementary material for: Actin’s functional “switch”: Constraining C-terminal conformational flexibility disrupts functionally important communication networks
Source: J Biol Chem. 2026 Apr 9;302(6):111446. doi: 10.1016/j.jbc.2026.111446 (PMC13156746; doi:10.1016/j.jbc.2026.111446)
Supplement: Supporting Information [file mmc1.docx]

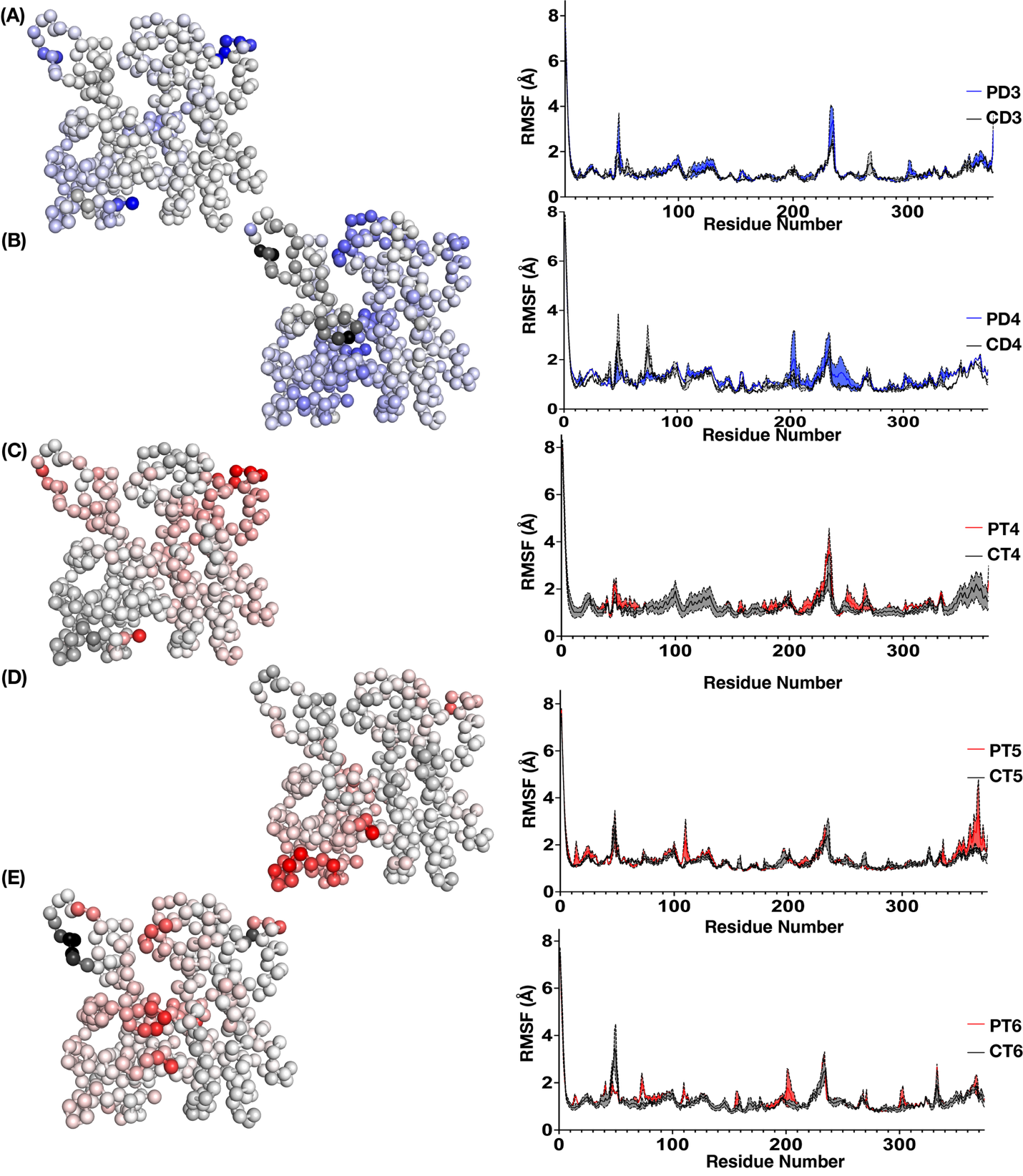


**Supporting Figure 1-Structural changes in other protomers:** Differences in actin’s structure between PBM-crosslinked and un-crosslinked protomers was analyzed. **(A)** ΔRMSF values (Control – PBM) were calculated for central dimer protomers (protomer 3 as shown in **Figure 1**) and projected onto the structure of an actin protomer. Negative values (PBM > Control, coloured blue) indicate increased residue flexibility in the PBM structure, while positive values (Control > PBM, coloured black) indicate increased flexibility in the control structure. Average RMSF values used for structural projections were plotted for CD3 (black) and PD3 (blue) protomers. **(B)** ΔRMSF values (Control – PBM) were calculated for dimer protomer 4 (as shown in **Figure 1**) and projected onto the structure of an actin protomer. Negative values (PBM > Control, coloured blue) indicate increased residue flexibility in the PBM structure, while positive values (Control > PBM, coloured black) indicate increased flexibility in the control structure. Average RMSF values used for structural projections were plotted for CD4 (black) and PD4 (blue) protomers. **(C)** ΔRMSF values (Control – PBM) were calculated for central trimer protomers (protomer 4 as shown in **Figure 1**) and projected onto the structure of an actin protomer. Negative values (PBM > Control, coloured red) indicate increased residue flexibility in the PBM structure, while positive values (Control > PBM, coloured black) indicate increased flexibility in the control structure. Average RMSF values used for structural projections were plotted for CT4 (black) and PT4 (red) protomers. **(D)** ΔRMSF values (Control – PBM) were calculated for trimer protomer 5 (as shown in **Figure 1**) and projected onto the structure of an actin protomer. Negative values (PBM > Control, coloured red) indicate increased residue flexibility in the PBM structure, while positive values (Control > PBM, coloured black) indicate increased flexibility in the control structure. Average RMSF values used for structural projections were plotted for CT5 (black) and PT5 (red) protomers. **(E)** ΔRMSF values (Control – PBM) were calculated for trimer protomer 6 (as shown in **Figure 1**) and projected onto the structure of an actin protomer. Negative values (PBM > Control, coloured red) indicate increased residue flexibility in the PBM structure, while positive values (Control > PBM, coloured black) indicate increased flexibility in the control structure. Average RMSF values used for structural projections were plotted for CT6 (black) and PT6 (red) protomers.


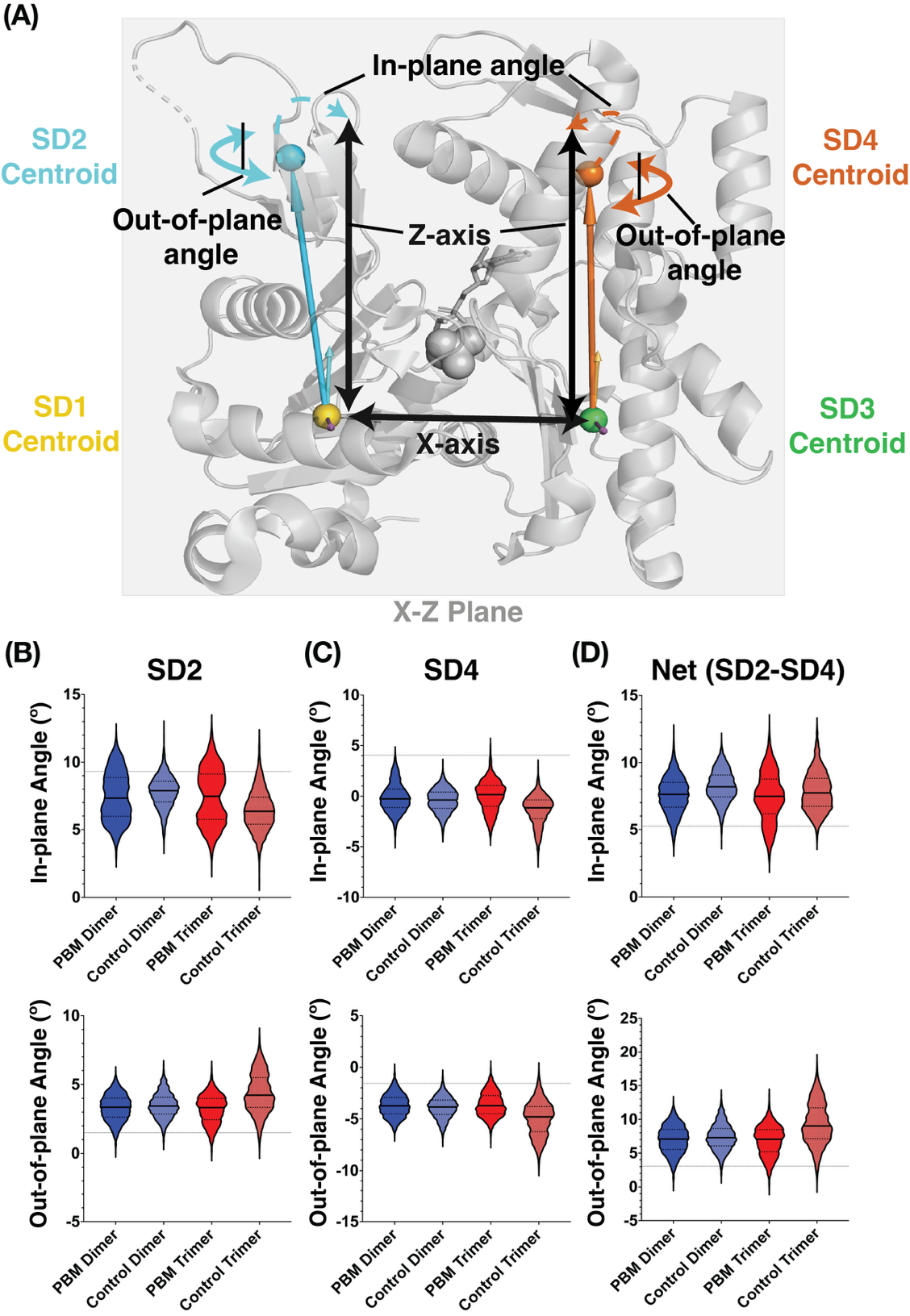


**Supporting Figure 2-Actin domain tilt**: The relative tilt of actin’s two major domains (SDs 1+2, SDs 3+4) was measured. **(A)** An axis system for each time point was defined, where the X-axis connects the centroids (mean position of all residues) of SDs 1 and 3, Z-axis perpendicular to the X-axis in the direction of SD2 (up/down), and the Y-axis perpendicular to these axes (in/out of the screen). Within this axis system, the XZ-plane was defined to represent the ‘face’ of the actin protomer. The angles of vectors connecting SDs 1-2 (cyan) and 3-4 (orange) were measured relative to the Z-axis. The left-right angles of these vectors (ie within the XZ-plane) is referred to as the ‘in-plane’ angle (positive=left), while the forwards-backwards angles of these vectors (within the YZ-plane) is referred to as the ‘out-of-plane’ angle (positive=’out’ of the screen). One subunit of PDB 8A2S was used to represent the axis system. **(B)** The in- and out-of-plane angles for the SD1-2 vector was plotted for each system across all simulation time points. The horizontal dotted lines (in-plane=9.304º, out-of-plane=1.497º) indicate the angle of this vector in the reference structure, PDB 8A2S. Within the violin plots, mean values are represented by solid lines and quartiles by dotted lines. **(C)** The in- and out-of-plane angles for the SD3-4 vector was plotted for each system across all simulation time points. The horizontal dotted lines (in-plane=4.405º, out-of-plane=-1.57º) indicate the angle of this vector in the reference structure, PDB 8A2S. Within the violin plots, mean values are represented by solid lines and quartiles by dotted lines. **(D)** The net domain tilt was measured as the difference in angles between the SD1-2 and SD3-4 vectors. The horizontal dotted lines (in-plane=5.259º, out-of-plane=3.067º) indicate net-tilt in the reference structure, PDB 8A2S. Within the violin plots, mean values are represented by solid lines and quartiles by dotted lines.


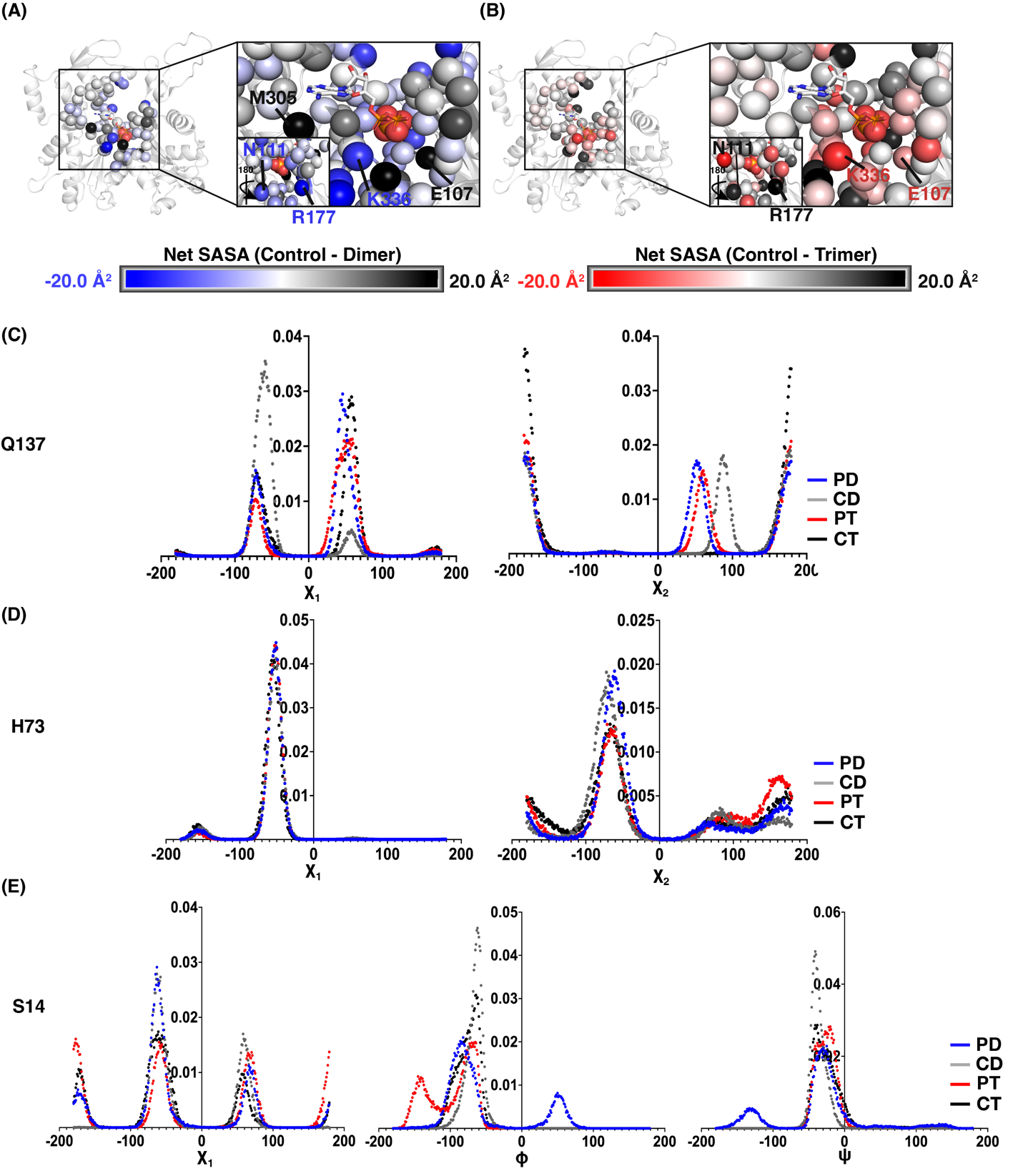


**Supporting Figure 3-Changes to nucleotide cleft architecture: (A)-(B)** Solvent accessible surface area (SASA) was calculated for each residue in the nucleotide binding cleft. Differences in SASA (Control – PBM) were calculated and projected onto the structure. Negative values (PBM > Control, blue for Dimer, red for Trimer) indicate residues with greater SASA in the PBM structure, relative to control. Positive values (Control > PBM, coloured black) indicated residues with greater SASA in the Control structure, relative to PBM. Residue alpha-carbons are represented as spheres. **(C)-(E)** Histograms of dihedral angles are shown for key residues regulating nucleotide dynamics (Q137, H73, S14) in the PD (blue), CD (grey), PT (red), and CT (black) systems.
